# Plasma arylsulfatase A levels are associated with cognitive function in Parkinson’s disease

**DOI:** 10.1007/s10072-022-06093-w

**Published:** 2022-04-29

**Authors:** Mingjian Li, Xiaoxue Shi, Jianjun Ma, Wenhua Sun, Zhidong Wang, Dongsheng Li, Jinhua Zheng, Zhenxiang Zhao, Qi Gu, Siyuan Chen

**Affiliations:** 1 Department of Neurology, Henan University People’s Hospital, Zhengzhou, China; 2 Department of Neurology, Henan Provincial People’s Hospital, Zhengzhou, China; 3 Department of Neurology, Zhengzhou University People’s Hospital, Zhengzhou, China

**Keywords:** Parkinson’s disease, Arylsulfatase A, α-Synuclein, Cognitive function

## Abstract

**Background:**

Arylsulfatase A (ARSA), a lysosomal enzyme, has been shown to inhibit the aggregation and propagation of α-synuclein (α-syn) through its molecular chaperone function. The relationship between ARSA levels and Parkinson’s disease (PD) in the Chinese Han population remains controversial, and few quantitative research studies have investigated the relationship between plasma ARSA levels and PD.

**Objectives:**

The purpose of this study was to investigate the relationships between ARSA levels and cognitive function in PD patients and to evaluate the association of ARSA and α-syn levels with nonmotor symptoms.

**Methods:**

Enzyme-linked immunosorbent assay (ELISA) was used to measure the plasma ARSA and α-syn levels in 50 healthy controls, 120 PD patients (61 PD patients with no cognitive impairment (PD-NCI) and 59 PD patients with cognitive impairment (PD-CI)). Motor symptoms and nonmotor symptoms (cognitive function, Unified Parkinson’s Disease Rating Scale (UPDRS) score, depression, anxiety, constipation, olfactory dysfunction, sleep disruption, and other symptoms) were assessed with the relevant scales. The Kruskal–Wallis *H* test was used for comparison between groups, and *Pearson/Spearman analysis* was used for correlation analysis.

**Results:**

The plasma ARSA concentrations were lower in the PD-CI group than in the PD-NCI group. The plasma α-syn levels in the PD-CI group were higher than those in the healthy control group, and the plasma ARSA levels were correlated with the Mini-Mental State Examination (MMSE scores) and Hoehn and Yahr (H-Y) stage.

**Conclusion:**

We used a quantitative assessment method to show that low plasma ARSA levels and high α-syn levels are related to cognitive impairment in PD patients. Plasma ARSA levels gradually decrease with PD progression.

## Introduction

Parkinson’s disease (PD) is a degenerative disease of the central nervous system that is commonly observed in middle-aged and elderly people and has been proven to be related to the deposition of Lewy bodies. α-Synuclein (α-syn) is the main component of Lewy bodies, and therefore, α-syn is closely related to PD. Recent studies have shown that arylsulfatase A (ARSA) is associated with PD and may participate in the aggregation and transmission of α-syn through its molecular chaperone function [1]. PD combined with cognitive impairment (PD-CI) is common in the middle and late stages of PD, and 80% of PD patients will eventually develop Parkinson’s disease dementia (PDD) [2]. Plasma ARSA levels are associated with motor symptoms and decrease with disease progression. PDD seriously affects the life and work of PD patients and increases disability and mortality rates. In addition, plasma ARSA levels are associated with cognitive function [3]. Some studies [4, 5] have not identified PD-related ARSA variants in the Chinese Han population through gene-sequencing analysis. At present, research results on the relationship between ARSA levels and PD are not consistent. To date, no clear and effective biological markers of PDD have been identified except that the amyloid β level of PDD patients is lower than that of PD patients with normal cognitive function [6, 7]. Therefore, this study quantitatively assessed the relationship between plasma ARSA concentrations and cognitive impairment in PD patients and measured the plasma concentrations of α-syn and their correlation with PD nonmotor symptoms.

## Materials and methods

### Participants

A total of 120 patients with primary PD diagnosed at Henan Provincial People’s Hospital were consecutively enrolled in this study. Patients were diagnosed by two experienced neurologists according to the clinical diagnostic criteria of the International Parkinson’s Disease and Movement Disorders Association [8]. Based on their MMSE scores [9], 59 patients were classified as having PD-CI, and 61 patients were classified as having PD with no cognitive impairment (PD-NCI). The exclusion criteria were as follows: (1) patients with secondary PD or Parkinson’s superposition syndrome and (2) patients with severe liver or kidney dysfunction, malignant tumors, or other serious primary diseases. Additionally, 50 age- and sex-matched healthy controls (HCs) were recruited from the Physical Examination Department of Henan People’s Hospital. The inclusion criteria were as follows: (1) no history of nervous system disease or mental illness and (2) no history of diabetes, hypertension, cardiovascular or cerebrovascular diseases, or systemic diseases. This study was performed in accordance with the principles of the Declaration of Helsinki and was approved by the Ethics Committee of the Henan Provincial People’s Hospital. The purpose and nature of the study were explained to all the subjects, and all the participants signed a written informed consent form.

### Clinical assessment

General clinical data, such as name, sex, age, age at onset, mode of onset, course of the disease, years of education, occupation, family history of medication, and other diseases, were collected. Based on a formula, the drugs and corresponding doses currently taken by the patients were transformed to a levodopa equivalent daily dose (LEDD). All patients were clinically evaluated in the nondrug phase. The Unified Parkinson’s Disease Rating Scale (UPDRS) and the modified Hoehn and Yahr (H-Y) scale were used to evaluate the severity of the disease. The following scales were used to assess nonmotor symptoms (Table 1).

**Table 1:** The clinical assessment of nonmotor symptoms

Nonmotor symptoms	Clinical assessment
Cognitive function	The Mini-Mental State Examination (MMSE)*
Anxiety	The 14-item Hamilton Rating Scale for Anxiety (HAMA-14)
Depression	The 17-item Hamilton Rating Scale for Depression (HAMD-17)
Sleep quality	The Pittsburgh Sleep Quality Index (PSQI)
Excessive daytime sleepiness	Epworth Sleepiness Scale (ESS)
Hyposmia	Argentine hyposmia scale (AHRS)
Constipation	Constipation scale
Nonmotor symptoms	Non-Motor Symptom Scale (NMSS)

**Fig. 1:**
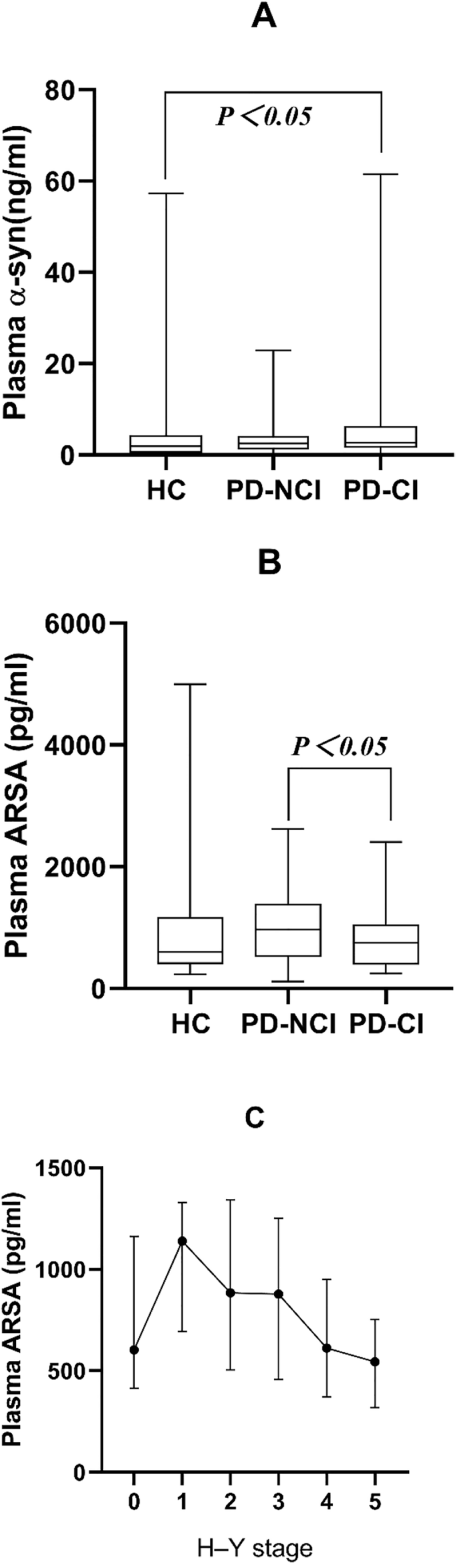
**A** The plasma α-syn level in the PD-CI group was higher than that in the control group (*P* = 0.04). **B** The plasma ARSA level in the PD-NCI group was higher than that in the PD-CI group (*P* = 0.021). **C** Among the H-Y stages, 0 represents the control group. The plasma ARSA level of PD patients was higher than that of healthy controls, and with the progression of the disease, the plasma ARSA level exhibited a gradual significant decrease (*P* = 0.07)

*The MMSE is divided into eight sub-items [10]: orientation to time and place (10 points); immediate registration (3 points); divided attention (5 points); delayed recall (3 points); language (4 points); following a three-step command (3 points); writing a sentence (1 point); and copying a Fig. (1 point)

### Blood sample collection and measurement of plasma ARSA and α-syn levels

All the subjects provided fasting blood samples in the morning that were collected in a tube coated with anticoagulants and centrifuged at 2500 r/min at 4 °C for 15 min. Then, the plasma was separated and stored in a – 80 °C freezer until analysis. The concentrations of ARSA (catalog number: MM-1380HE; Jiangsu Enzyme Industrial Co., Ltd., Jiangsu, China) and α-syn (catalog number: ZC-32191; Shanghai Zhuocai Biotechnology Co., Ltd., Shanghai, China) were measured with the sandwich enzyme-linked immunosorbent assay (ELISA) method. A standard curve generated with purified ARSA was used to calculate the ARSA concentration of each sample based on the absorbance at *λ* = 450 nm. The standard curves had ranges of 25–850 pg/ml and 1.25–40 ng/ml, respectively. The intra-assay coefficients of variation in the ARSA (human) ELISA kit/α-syn ELISA kit were both calculated to be 10%. All samples were analyzed in duplicate and processed in the first freeze–thaw cycle.

### Statistical analysis

SPSS 25.0 (IBM, USA) and GraphPad Prism 8.0 (GraphPad Prism Software, Inc., San Diego, CA, USA) were used for statistical analysis and plotting. Quantitative data with a normal distribution are expressed as the mean ± standard deviation (*X* ± *s*). A *t* test was used for comparisons between the two groups. Pearson’s chi-square test was used to compare the distributions of categorical variables between groups. Data that were not normally distributed are presented as the median (quartile range). Variables that did not fit the normal distribution were assessed using the Mann–Whitney *U* test (two groups) or the Kruskal–Wallis (KW) rank-sum test (three groups). Spearman’s correlation analysis was used to analyze the relationship between ARSA levels and nonmotor symptoms and MMSE sub-items. The significance level of the test was set to *α* = 0.05.

## Results

The central line in each box indicates the median, the box edges mark the first and third quartiles, and the limits of the vertical lines show ranges. In Fig. 1C, the middle point represents the median, and the vertical lines represent the first and third quartiles.

1. Demographic data and plasma ARSA levels of HCs, PD-CI patients, and PD-NCI patients
  There were no statistically significant differences in age or sex among the three groups. Moreover, there were no statistically significant differences in the course of the disease or total LEDD between the PD-CI and PD-NCI groups, but there were statistically significant differences in the MMSE scores between the two groups (*P* < 0.001) (Table 2). The difference in α-syn concentrations between the HC group and the PD-CI group was statistically significant (*P* = 0.015), and the concentration in the PD-CI group was higher than that in the control group. The differences in ARSA concentrations among the three groups were statistically significant (*P* = 0.035), and the plasma ARSA concentrations in the PD-NCI group were higher than those in the PD-CI group (*P* = 0.043) (Fig. 1A and B).
2. ARSA concentrations in relation to H-Y stage and MMSE scores
  ARSA concentrations were correlated with MMSE scores (*P* = 0.01) and the H-Y stage (*P* = 0.011) (Table 3). ARSA concentrations were positively correlated with MMSE scores, suggesting that when the patients’ MMSE scores were lower, the concentrations of ARSA were lower, which was consistent with the results shown in Table 3. ARSA concentrations were negatively correlated with the H-Y stage, as shown in Fig. 1C. With increases in the H-Y stage, ARSA concentrations showed a gradual significant decrease (*P* = 0.07). ARSA concentrations were not correlated with α-syn concentrations and had no significant correlations with HAMA, HAMD, constipation, sleep, smell, or nonmotor symptom total scores in PD patients.
3. Correlations between plasma ARSA levels and MMSE sub-item scores
  Plasma ARSA levels were positively correlated with the following sub-items of the MMSE: orientation to time and place, divided attention and delayed recall (*P* < 0.05) (Table 4).

**Table 2:** Demographic data of healthy controls and Parkinson’s disease (PD) patients

	HC (*n* = 50)	PD-NCI (*n* = 61)	PD-CI (*n* = 59)	*P* value
Age (years)	61.5 (56, 66.25)	63 (55.5, 67)	65 (62, 69)	0.06
Sex (male/female)	25/25	36/25	28/31	0.414
Age of onset (years)	NA	56 (49.5, 61)	60 (54, 64)	0.022
Disease duration (years)	NA	5 (3, 9)	6 (4, 8)	0.754
LEDD (mg)	NA	400 (200, 636.75)	450 (300, 600)	0.457
MMSE	NA	28 (27, 29)	22 (19, 24)	< 0.001

**Table 3:** Relationship between plasma ARSA levels and nonmotor symptoms

	Median (quartile range)/mean (standard deviation)	Pearson/Spearman rank	*P* values
Age (y)	64 (59.25, 68)	0.108	0.242
MMSE score	26 (22, 28)	0.234	0.01
α-syn (ng/ml)	2.64 (1.38, 5.17)	0.008	0.967
UPDRS score	68 (49, 89.75)	0.153	0.096
LEDD (mg/d)	427.5 (300, 600)	− 0.143	0.119
Disease duration (y)	5 (3.25, 8)	0.066	0.472
H–Y stage	2.75 (2, 3)	− 0.23	0.011
HAMA score	13.5 (9, 17)	0.117	0.205
HAMD score	12.36 (5.81)	0.06	0.518
Constipation scale score	5 (0, 7)	0.128	0.164
PSQI score	8 (5, 12)	0.054	0.559
ESS score	7 (3, 10)	0.165	0.072
AHRS score	20 (10, 24)	− 0.094	0.308
NMSS score	45 (28.25, 71)	− 0.086	0.352

**Table 4:** Correlations between ARSA and MMSE sub-item scores

Items	Median (quartile range)	Spearman rank	*P* values
Orientation to time and place (10 points)	10 (9, 10)	0.238	0.009
Immediate registration (3 points)	3 (3, 3)	-0.003	0.978
Divided attention (5 points)	4 (2, 5)	0.225	0.014
Delayed recall (3 points)	2 (1, 3)	0.186	0.042
Language (4 points)	4 (3, 4)	0.026	0.782
Following a three-step command (3 points)	3 (3, 3)	0.014	0.881
Writing a sentence (1 points)	1 (0, 1)	0.137	0.136
Copying a Fig. (1 points)	0 (0, 1)	0.099	0.281

## Discussion

At present, the etiology of PD is still unclear. Mitochondrial dysfunction, oxidative stress, disordered lipid metabolism, lysosomal dysfunction, excessive neuroinflammation, disordered microbiota, and other possible mechanisms may underlie the pathogenesis of PD [11]. The neuropathological characteristics of PD include the production of α-syn, which aggregates into Lewy bodies and accumulates in nerve cells; these α-syn aggregates are harmful to dopaminergic neurons in the central nervous system and may trigger the formation of toxic α-syn, which is passed from affected cells to neighboring cells, leading to cell death [12, 13]. In addition, α-syn is degraded via the proteasomal/autophagosomal pathways [14]. These pathways themselves can impair lysosomal function, and any damage to the clearance pathway may lead to further α-syn transmission [15], creating a vicious cycle. In addition, lysosomal dysfunction has been shown to lead to intercellular aggregation, diffusion, and transmission of α-syn [16].

Many recent studies [17, 18] have revealed a relationship between lysosomal storage disorders and common neurodegenerative diseases, the most common example of which is the relationship between GBA1 mutations and PD. Recessive mutations in the GBA gene, which encodes glucocerebrosidase, a lysosomal hydrolase, are responsible for Gaucher disease, and carriers of heterozygous GBA mutations have a significantly higher incidence of PD than the general population. However, a study [19] has shown that with or without mutations in the GBA or LRRK2 genes, lower glucocerebrosidase activity can be observed in PD patients than in healthy individuals. It has been speculated that glucocerebrosidase is a protective factor against PD. ARSA is a lysosomal hydrolase that converts thiaminic acid to galactosylceramide. Mutations in the ARSA gene often cause autosomal recessive lysosomal storage disorder. Allochromatic leukodystrophy is an autosomal lysosomal storage disorder [20]. ARSA has been shown to be a molecular chaperone of α-syn that inhibits α-syn secretion, aggregation, and transmission through its molecular chaperone function [1].

In this study, we found that there was no correlation between plasma ARSA and α-syn levels in PD patients. The reason may be that the plasma concentrations of ARSA and α-syn were measured in this study, and α-syn is present mainly in the cytoplasm; therefore, the binding of ARSA and α-syn also occurs mainly in the cytoplasm, and the effect may not be apparent in the plasma. ARSA-specific antibodies or normal IgG have been used as a negative control to conduct α-syn immunoprecipitation, and a large amount of ARSA was found in the cytoplasm and vesicles; this result indicated that ARSA can directly bind to α-syn, and most of the intermediate binding occurs in the cytoplasm. ARSA is widely expressed in brain and spinal cord cells, blood lymphocytes, and peripheral tissue cells, and can also be secreted by cells [21]. Binding and expression in plasma require further investigation [1]. However, the changes in plasma ARSA levels in PD and PDD patients suggest that ARSA in the plasma is indeed involved in the pathogenesis of PD.

Previous studies [5] have shown that the L300S heterozygous mutation of the ARSA gene may be a risk factor for PD; however, the N352S variant does not reduce the activity of the ARSA enzyme and acts as a molecular chaperone of α-syn to inhibit the aggregation and diffusion of α-syn, playing a protective role. A large genetic sequencing study [4] found that ARSA variants showed no association with PD susceptibility or cognitive impairment. In this study, we explored the correlation between plasma ARSA protein levels and PD course and cognitive impairment; this correlation may occur due to possible regulatory effects on ARSA translation, resulting in differences in the protein level. The small sample size in this study may also be the reason for the inconsistency with the above research results.

This study showed that the plasma ARSA levels in PD patients were related to the H-Y stage and that the ARSA concentrations of patients with early PD were higher than those of healthy controls. With the gradual progression of the disease, the ARSA concentrations gradually decreased. It can be speculated that ARSA may be a protective factor against PD and is related to degeneration of the substantia nigra and striatum. ARSA may compensate for the autophagic clearance of α-syn in neurons, which gradually becomes insufficient with the progression of the disease; thus, ARSA levels gradually decrease. In PD, one-third of patients with higher glucosidase levels had a longer course of the disease, which may be described as a milder disease, and the activity of glucosidase may serve as a marker of PD severity [19]. A study [3] pointed out that the ARSA levels in the body were related to the course of the disease and that the ARSA levels and duration of the disease showed an inverted U-shaped trend, reaching a peak approximately 2 years after onset, which is consistent with this study. Additionally, research [5] has identified ARSA genetic variations among the Han population in China and in PD patients, which means that PD patients with ARSA mutations are rarely identified; therefore, more large-scale case–control studies need to be conducted in familial and sporadic PD or other populations to determine whether the ARSA gene is associated with the incidence of PD [22].

In this study, the plasma ARSA concentrations were lower in the PD-CI group than in the PD-NCI group. In the correlation analysis, the ARSA concentrations were positively correlated with the MMSE scores. Consistent with this study, Lee J.S. et al. [1] confirmed that plasma ARSA concentrations were lower in PDD patients. Plasma ARSA levels were positively correlated with overall cognitive ability, total MMSE scores, and scores in various cognitive domains. In one study, mice were injected with lentiviral vectors expressing hemagglutinin- or green fluorescent protein-labeled ARSA cDNA, and a significant accumulation of ARSA activity was observed on both sides of the hippocampus, suggesting that ARSA is associated with cognitive function [23].

When we further investigated the correlations between the ARSA levels and MMSE sub-item scores, ARSA levels were found to be positively correlated with three sub-items: orientation to time and place, divided attention, and delayed recall. Studies have shown that among patients with mild cognitive impairment, cognitive impairment is mainly related to time and spatial orientation, attention and language [24]. In this study, the delayed recall was impaired in PD patients with low ARSA concentrations, which may have occurred because the sample population in this study included patients with mild cognitive impairment and dementia, and with the increase in the severity of cognitive impairment, the delayed recall was gradually impaired.

In this study, plasma α-syn concentrations were higher in the PD-CI patients than in the PD-NCI patients, and the levels in these patients were higher than those in the HCs, suggesting that higher plasma α-syn concentrations are associated with increasingly poor cognitive abilities. In addition, a recent study showed that a specific genetic haplotype in intron 4 in the SNCA gene was associated with the risk of PDD [25]. Studies have confirmed that high levels of α-syn in plasma are associated with cognitive impairment in PD patients [26, 27].

In conclusion, plasma ARSA levels are increased in the early stage of PD and decrease gradually with the progression of the disease. Low plasma concentrations of ARSA and high concentrations of α-syn are associated with cognitive impairment in PD patients, and plasma ARSA levels are negatively correlated with the H-Y stage. Based on this conclusion, ARSA may serve as a biomarker for the early diagnosis of PD and may be involved in the mechanism underlying the cognitive impairment of PD patients.
